# Race, Ethnicity, and Other Cultural Background Factors in Trials of Internet-Based Cognitive Behavioral Therapy for Depression: Systematic Review

**DOI:** 10.2196/50780

**Published:** 2024-02-01

**Authors:** Robinson De Jesús-Romero, Amani R Holder-Dixon, John F Buss, Lorenzo Lorenzo-Luaces

**Affiliations:** 1 Department of Psychological and Brain Sciences Indiana University - Bloomington Bloomington, IN United States; 2 Department of Psychiatry Indiana University School of Medicine Indianapolis, IN United States

**Keywords:** diversity, cognitive behavioral therapy, internet-based, depression, race, racial, ethnicity, culture, depressive, diverse, inclusive, inclusivity, DEI, diversity, equity, and inclusion, internet-based cognitive behavioral therapy, mental health, ethnic, cultures, culturally, review methods, review methodology, systematic, clinical trial, clinical trials, randomized controlled trial, randomized controlled trials, controlled trial, controlled trials, reporting, immigrant, migrant, migrants, immigrants, psychotherapy, underrepresented, underrepresentation, representation, mobile phone

## Abstract

**Background:**

There is a growing interest in developing scalable interventions, including internet-based cognitive behavioral therapy (iCBT), to meet the increasing demand for mental health services. Given the growth in diversity worldwide, it is essential that the clinical trials of iCBT for depression include diverse samples or, at least, report information on the race, ethnicity, or other background indicators of their samples. Unfortunately, the field lacks data on how well diversity is currently reported and represented in the iCBT literature.

**Objective:**

Thus, the main objective of this systematic review was to examine the overall reporting of racial and ethnic identities in published clinical trials of iCBT for depression. We also aimed to review the representation of specific racial and ethnic minoritized groups and the inclusion of alternative background indicators such as migration status or country of residence.

**Methods:**

Studies were included if they were randomized controlled trials in which iCBT was compared to a waiting list, care-as-usual, active control, or another iCBT. The included papers also had to have a focus on acute treatment (eg, 4 weeks to 6 months) of depression, be delivered via the internet on a website or a smartphone app and use guided or unguided self-help. Studies were initially identified from the METAPSY database (n=59) and then extended to include papers up to 2022, with papers retrieved from Embase, PubMed, PsycINFO, and Cochrane (n=3). Risk of bias assessment suggested that reported studies had at least some risk of bias due to use of self-report outcome measures.

**Results:**

A total of 62 iCBT randomized controlled trials representing 17,210 participants are summarized in this study. Out of those 62 papers, only 17 (27%) of the trials reported race, and only 12 (19%) reported ethnicity. Reporting outside of the United States was very poor, with the United States accounting for 15 (88%) out of 17 of studies that reported race and 9 (75%) out of 12 for ethnicity. Out of 3,623 participants whose race was reported in the systematic review, the racial category reported the most was White (n=2716, 74.9%), followed by Asian (n=209, 5.8%) and Black (n=274, 7.6%). Furthermore, only 25 (54%) out of the 46 papers conducted outside of the United States reported other background demographics.

**Conclusions:**

It is important to note that the underreporting observed in this study does not necessarily indicate an underrepresentation in the actual study population. However, these findings highlight the poor reporting of race and ethnicity in iCBT trials for depression found in the literature. This lack of diversity reporting may have significant implications for the scalability of these interventions.

## Introduction

### Background

In 2020, approximately 34% of the US population identified themselves as belonging to racial minoritized groups, and 19% identified as members of ethnic minoritized groups [1]. Following the terminology used by other scholars [2], we employ the term “minoritized” rather than “minorities” to emphasize that these individuals’ experiences are not intrinsic qualities of statistically small groups; instead, they are the result of dominant groups subordinating and, consequently, “minoritizing” them. The proportion of individuals belonging to a racial or ethnic minoritized group in the United States is expected to continue growing [3]. This increase in the proportion of minoritized groups is also expected in other countries. There are currently an estimated 272 million immigrants worldwide, with 82 million residing in Europe, 59 million in North America, and 49 million in Northern Africa and Western Asia [4]. Therefore, it is essential that mental health research responds to this increase in racial and ethnic diversity.

Mental health is one of the leading causes of disability in the United States and worldwide [5]. Among mental disorders, depression is the leading cause of significant disability [6] with an estimated economic burden of around US $210.5 billion per year in the United States alone [6]. Thus, given that mental health is strongly correlated with racial-ethnic identity and with other factors that are themselves linked to mental health (eg, socioeconomic status), there is a strong imperative for mental health research to represent the racial-ethnic diversity in our populations [7-9].

### Current Reporting on Race and Ethnicity

Current research in mental health continues to use primarily non-Hispanic White populations, which fails to reflect the demographic makeup of countries worldwide. For example, in a study by Mak and colleagues [10], they reviewed 379 National Institute of Mental Health–funded clinical trials for various mental health disorders published between 1995 and 2004 to investigate how many trials reported sex, race, and ethnicity. They found that 91.6% of the National Institute of Mental Health–funded published trials reported sex. However, only 47.8% included race or ethnicity in their demographics, and 25.6% had incomplete race or ethnicity information.

Since then, the overall pattern of reporting demographic information has improved slightly. A more recent meta-analysis by Polo and colleagues [11] examines the trends in reporting and representation of racial-ethnic diversity in randomized controlled trials (RCT) of psychotherapy for depression for over 36 years. They found that reporting of racial-ethnic group membership increased from 16% to 55% during this time. This increase was attributed to the introduction of new guidelines aimed at increasing gender, race, and ethnicity reporting on RCTs. These guidelines included the National Institutes of Health Guidelines on the Inclusion of Women and Minorities as Subjects in Clinical Research in 1994 [12], the CONSORT (Consolidated Standards of Reporting Trials) [13] in 2001, and the American Psychological Association Publications and Communications Board Working Group on Journal Article Reporting Standards (JARS) in 2008. However, the reporting of treatment effects by ethnic groups remained at a low at 2.1% [11]. Additionally, only non-Hispanic Black and Latino individuals were represented significantly more than in previous years. Asian Americans, multiracial individuals, Native Americans or Native Alaskans, and Native Hawaiians or Pacific Islanders were still underrepresented in the literature, with no significant change across time [11] despite evidence that participants are willing to engage in web-based health-related research [14].

### Depression and Health Disparities

Existing research on depression and race-ethnicity presents a somewhat complex picture. In the United States, apart from Native Americans, who have the highest rates of depression, minoritized groups tend to have lower rates of depression than non-Hispanic White individuals [15]. However, minoritized individuals may be at a higher risk for more severely debilitating depression when compared to non-Hispanic White individuals [16]. Additionally, the costs associated with treating depression may impact racial groups differently. For example, those who are middle class and Black tend to encounter more obstacles to upward mobility (ie, moving from one social class to another) and are more susceptible to downward mobility, which may impede their access to mental health care [17,18]. In 2020, 53% Black individuals experienced worse access to care than White individuals. Similarly, 29% of Asian individuals had worse access to care, while American Indian or Alaska Native individuals presented the largest disparity with 50% receiving worse access to care than White individuals. Native Hawaiian and Other Pacific Islander individuals, however, experienced the same level of access to care as White individuals [19].

Furthermore, substantial barriers in access to mental health care tend to affect minoritized racial-ethnic groups more than nonminoritized groups. For example, there is evidence that interpersonal factors (eg, stigma and personal shame), sociocultural factors (eg, fear of negative evaluation by family or peers, preference for traditional coping strategies such as withdrawal and “accepting fate”), and systemic factors (eg, lack of culturally centered clinical environments and culturally responsive services and language barriers) can make it difficult for members of racial-ethnic minoritized groups to access mental health care [20]. These barriers may be particularly pronounced in face-to-face mental health treatment settings, where patients need to engage fully with another person for it to be successful; however, innovative internet-based delivery methods (eg, internet-based cognitive behavioral therapy [iCBT] self-help) may reduce barriers to treatment by making it more accessible [21]. Moreover, there is evidence that shows that Latinx and non-Hispanic Black individuals may be willing to engage in smartphone-based interventions and bibliotherapy, respectively [22,23].

In addition to race and ethnicity, migration status is another crucial sociodemographic factor associated with depressive and anxious symptoms. For example, in a study involving 37,076 individuals from 20 European countries, first-generation migrants exhibited higher levels of depression, with rates significantly elevated for those born outside Europe [24]. Research on refugees resettled in high-income countries indicates that they experience increased rates of anxiety and depression compared to the general population [25]. These findings suggest that aspects such as migration status, country of origin, refugee status, and other related demographics play a significant role in predicting depression and anxiety symptoms, emphasizing the importance of providing mental health services to cater to these populations.

### iCBT for Depression

One of the most studied and promising internet-based interventions for depression is iCBT. iCBT involves the provision of self-help materials (eg, websites, apps, and videos) that impart psychoeducation and teach skills that individuals can use to manage their symptoms. iCBT is usually delivered in 1 of 2 formats: guided or unguided. In guided self-help, individuals access self-help material and are assisted by a trained mental health professional or paraprofessional [26]. In unguided self-help, individuals access materials independently, without assistance or support. Both guided and unguided iCBT treatments have been shown to be more efficacious than waiting list controls [27].

Although unguided forms of self-help are more scalable and easier to access, guided self-help has been shown to be more effective than unguided self-help and as effective as face-to-face treatment [27]. Individuals may also adhere to it more closely [28]. Considering the wide support for the efficacy of iCBT, this format has the potential to reduce the public health burden of untreated depression, especially among individuals from a racial-ethnic minoritized group [21,29]. Given the potential for iCBT to reach minoritized communities, it is important to understand the reporting and representation of race-ethnicity in iCBT studies. If individuals from racial-ethnic minoritized groups are being underreported, it would be difficult to determine how well our current interventions meet the needs of these individuals.

For these reasons, we explored the reporting and representation of racial-ethnic diversity in clinical trials of iCBT for depression. Our first aim was to examine the overall reporting of individuals from racial-ethnic minoritized groups in published RCTs of iCBT for depression. Our second aim was to explore the representation of specific racial-ethnic minoritized groups in RCTs of iCBT for depression. To achieve these aims, we conducted a systematic review of RCTs of iCBT for depression and examined the reporting of race-ethnicity along with the representation (ie, the sample composition) [30-90]. We also explored the reporting of other background factors (eg, migration status).

## Methods

### Search Strategies

To identify RCTs, we employed a 2-pronged search strategy. First, we searched METAPSY, a database of randomized clinical trials for depression created by Cuijpers [91] and colleagues [92]. The public version of the database covers a search of trials between January 1, 1966, and January 2018, including Risk of Bias (ROB) assessments. We obtained an updated database version from one of the METAPSY lead researchers (P Cuijpers), which included studies up to January 1, 2021. The database of 763 studies was created from a search on PubMed, PsycINFO, Embase, and the Cochrane Library. The search was performed using terms involving psychotherapy (eg, “psychotherapy” and “cognitive-behavioral therapy”) and depression (eg, “depressive symptoms” and “major depression”). An example of the search string for PubMed can be found in Multimedia Appendix 1. Studies were included if they were an RCT in which a psychotherapy condition was compared to another. Control conditions included waiting list, treatment as usual, pill placebo, pharmacotherapy, alternate therapy delivery modalities (eg, single vs group therapy), or any other active control condition. Exclusion criteria included (1) no statement of randomization; (2) depression not being an inclusion criterion; (3) not being focused on acute treatment (eg, maintenance or relapse prevention); (4) studies with children or adolescents; (5) dissertation studies; (6) studies in which the depression was not the target of treatment; (7) if effect sizes could not be calculated; and (8) studies in languages other than English, Spanish, German, or Dutch. METAPSY contains a risk-of-bias assessment using the risk-of-bias assessment tool from the Cochrane Collaboration for papers published before 2018 [92]. Two raters (ARH-D and RDJ-R) examined the titles and abstracts of the 763 studies identified in METAPSY to decide whether they were relevant for review.

To obtain more recent papers, we used the same search string used for METAPSY [92] with the addition of a search term for internet-based studies to search Cochrane, PubMed, PsycINFO, and Embase for papers published between January 1, 2021, and July 18, 2022. An example search string for PubMed is available in Multimedia Appendix 2. This second search yielded an additional 2159 studies, of which 590 were duplicates. We also identified an additional paper by cross-referencing a study protocol. Further, 4 raters used a randomized, counterbalanced design to review the titles and abstracts of the remaining 1570 studies (ARH-D, JFB, LL-L, and RDJ-R). Disagreements between the raters were resolved by consensus.

**Figure 1:**
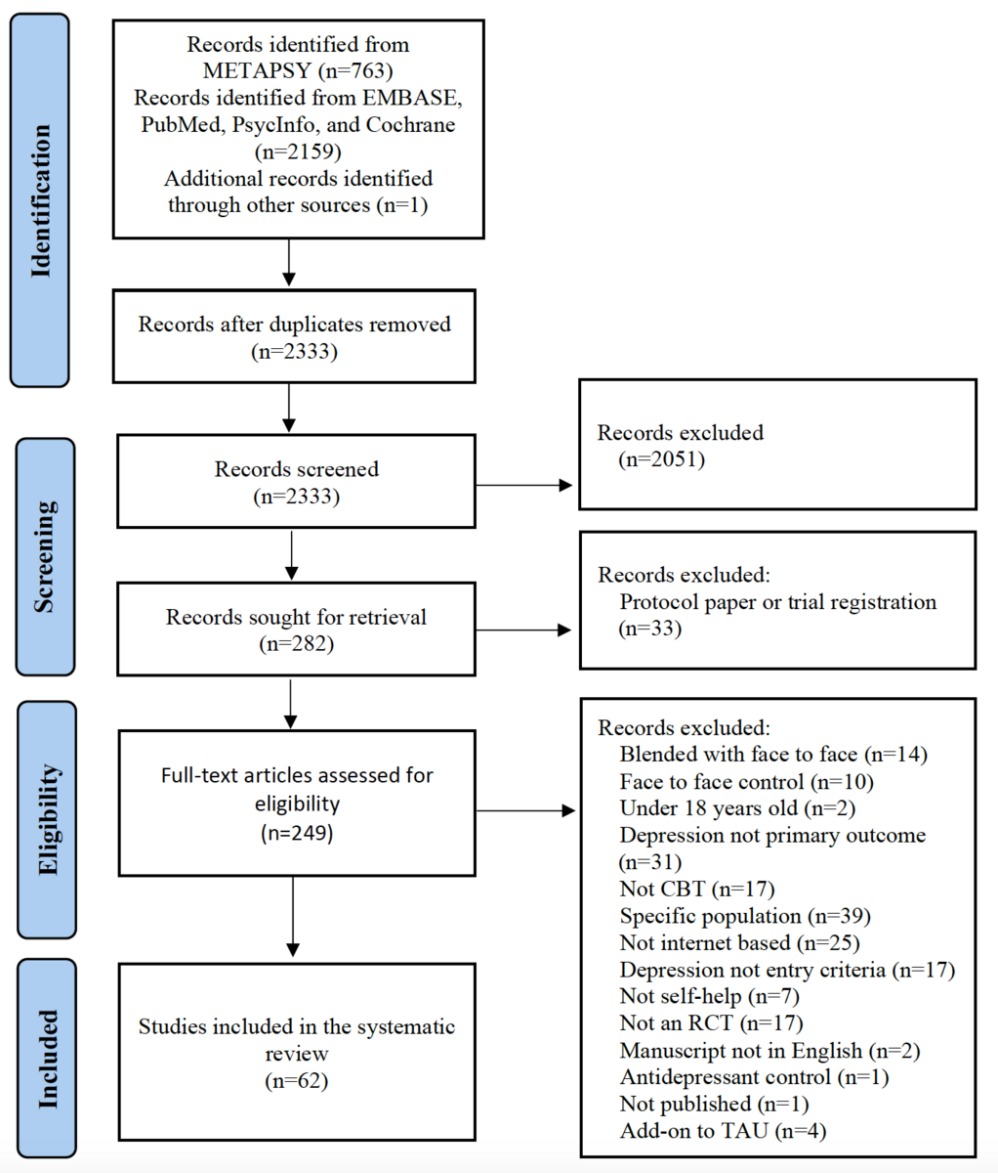
Flow diagram of papers at each stage of the screening process. CBT: cognitive behavioral therapy; RCT: randomized controlled trial; TAU: treatment as usual.

We included randomized controlled trials in which iCBT was compared to a waiting list, care-as-usual, active control, or another iCBT treatment (eg, 2 different iCBTs or unguided vs guided iCBT). The included papers must have also focused on acute treatment (eg, 4 weeks to 6 months) of depression, delivered via the internet on a website or a smartphone app as guided or unguided self-help iCBT. In addition to the exclusion criteria used for METAPSY [91,92], we also excluded studies if the (1) interventions were not delivered via the internet, and (2) interventions focused on specific medical or psychiatric subpopulations (eg, individuals with cannabis use disorder). We excluded studies that used specific subpopulation samples because we aimed to study the general reporting and representation of race-ethnicity in trials of iCBT for depression [93,94]. Additionally, we concentrated on studies that examined depression to prevent overlapping treatment outcomes (eg, chronic pain, sleep, and biases due to the specificity of the pool of participants) [95]. Of the 2333 studies, 282 were identified as relevant to self-help iCBT for depression. We then examined the full text of these studies and excluded those that did not meet our inclusion criteria. See Figure 1 for the PRISMA (Preferred Reporting Items for Systematic Reviews) flowchart. We assessed the ROB for 20 papers that did not already have an ROB assessment in the public version of the METAPSY database, 17 that were provided in the METAPSY update and 3 we found in our systematic search (Multimedia Appendix 3).

### Coding

Coders rated study characteristics, trial design aspects, intervention type, and control group. They also coded various study-level features, including year of publication, whether the studies used guided or unguided iCBT and the country from which the study was sampled. For our primary question of interest, we first coded whether studies included race or ethnicity as “1” if they did or “0” if they did not. Additionally, when studies reported race or ethnicity, we recorded the number of participants in the sample that belonged to the different racial and ethnic groups. We then calculated the proportion of minoritized group members in the sample for the studies that reported overall race or ethnicity. Finally, given that we included studies conducted outside the United States, where there may be different conceptualizations of race and ethnicity, we also recorded other alternative variables indicative of participant background, including migration status, country of birth, country of residence, nationality, or native language. Agreement between raters was based on consensus, with disagreements being resolved by the principal investigator (LL-L).

## Results

### Study Selection and Characteristics

In total, 62 papers met the criteria of an RCT of iCBT for depression in adults. Some studies used samples from multiple countries or reported multiple arms, therefore, there are more interventions and control conditions than the number of studies (ie, 62). These studies included a total of 64 interventions of which 41 (64%) included guided self-help, and 23 (36%) included unguided self-help. Regarding control groups, 34 (52%) out of the 65 control groups included were compared to waiting lists, 12 (18%) to treatment as usual, 12 (18%) to active controls, and 7 (11%) used an internet-based active treatment comparator (ie, guided or unguided iCBT). The studies drew on samples from 13 countries: United States (n=17), Germany (n=12), Australia (n=10), Sweden (n=10), the Netherlands (n=6), China (n=2), Finland (n=2), United Kingdom (n=2), Canada (n=1), Colombia (n=1), Ireland (n=1), New Zealand (n=1), and Switzerland (n=1). The 62 papers included in this study had a total sample of 17,210 participants. ROB information is available in Multimedia Appendix 3 and suggests that reported studies had at least some ROB. However, most of this bias was due to self-reporting as an outcome measure. In the other ROB domains, 14 (70%) out of 20 of studies had low risk concerning missing outcome data, while 18 (90%) out of 20 of studies showed low risk for deviation from intended interventions.

### Reporting of Race and Ethnicity

Of the 62 papers, only 17 (27%) reported race, and 12 (19%) reported ethnicity. Despite this overall low reporting rate, we observed that reporting differed markedly by the country sampled in the study (Fisher exact test *P*<.001). Of the studies conducted in the United States (n=17), almost all included race (n=15, 88%). Outside of the United States, only 2 studies conducted in the United Kingdom (n=1) and Australia (n=1) reported race. RCTs using samples from Germany, the Netherlands, Sweden, Ireland, Switzerland, China, Finland, Canada, Colombia, and New Zealand did not report race. Similarly, ethnicity was only reported in 12 studies, 4 of which grouped ethnicity with race. A fifth trial described reporting ethnicity, but it combined racial and ethnic categories; therefore, it was labeled as reporting both race and ethnicity. Additionally, 2 of them were conducted specifically on a minoritized group. The results are summarized in Table 1. Translating the reporting numbers to individuals, of the 17,210 participants in all these RCTs, 13,587 (78.9%) had no reported race, and 14,810 (86.1%) had no reported ethnicity.

**Table 1:** Number of published randomized controlled trials of internet-based cognitive behavioral therapy that reported race or ethnicity, by reported source country (N=62).

Country	Papers	Reported race, n (%)	Report ethnicity, n (%)
United States	17	15 (88)	9 (53)
Germany	12	0 (0)	0 (0)
Australia	10	1(10)	1 (10)
Sweden	10	0 (0)	0 (0)
Netherlands	6	0 (0)	1 (17)
China	2	0 (0)	0 (0)
Finland	2	0 (0)	0 (0)
United Kingdom	2	1 (50)	1 (50)
Canada	1	0 (0)	0 (0)
Colombia	1	0 (0)	0 (0)
Ireland	1	0 (0)	0 (0)
New Zealand	1	0 (0)	0 (0)
Switzerland	1	0 (0)	0 (0)

### Representation of Individuals From Racial and Ethnic Minoritized Groups

Of the 17 papers that reported race, 4 (24%) categorized groups into “minority” versus “non-minority” or “White” versus “other.” The other papers included the following groups on their breakdown: American Indian or Alaskan Native (n=3); Asian (n=8); Black or African American (n=12); Middle Eastern or North African (n=1); multiracial (n=8); Native American (n=1); other (n=8) which included “not specified,” “declined to answer,” and not reported as White; Pacific Islander (n=3); unknown (n=18); and White (n=16) (see Table 2).

**Table 2:** Total number of participants by race for trials of internet-based cognitive behavioral therapy for depression that reported race (N=3623).

Race	Participants, n (%)
AIAN^a^	14 (0.4)
Asian	209 (5.8)
Black	274 (7.6)
MENA^b^	1 (0)
Multiple	143 (3.9)
Native American	3 (0.1)
Not specified	75 (2.1)
Other	76 (2.1)
Pacific Islander	6 (0.2)
Unknown	106 (2.9)
White	2716 (74.9)

^a^AIAN: American Indian or Alaskan Native.

^b^MENA: Middle Eastern or North African.

The papers that reported ethnicity (Table 3) reported the following ethnic groups: American (n=1), Chinese (n=1), European (n=1), Hispanic or Latino (n=9), other (n=1), Turkish (n=1), and White British (n=1). Many papers specified ethnicity for minoritized member groups (eg, Hispanic) but not for majority members (eg, non-Hispanic).

**Table 3:** Total number of participants by ethnicity for trials of internet-based cognitive behavioral therapy for depression that reported ethnicity (N=2496).

Ethnicity	Participants, n (%)
American	2 (0)
Chinese	148 (5.9)
European	11(0)
Hispanic	281 (11.3)
Not specified (eg, reported ethnicity for some but not other individuals)	1886 (75.6)
Other	9 (0)
Turkish	96 (3.8)
White British	63 (2)

### Representation of Race in iCBT Trials in the United States

Given that reporting of race was highest in the United States, we explored the representation of individuals from racial-ethnic minoritized groups in the 15 RCTs from that country that reported race (n=3354). A total of 2568 out of 3354 (76.6%, 95% CI 75.1-78.0) of individuals in these samples were identified as White. By way of comparison, 61.6% of adults in the United States were reported as being White in the US census [1]. These rates are significantly different (χ^2^_1_=316.9, *P*<.001). Extrapolating the number of individuals that would be expected to be White based on the 12-month prevalence of depression reported in the latest epidemiological study (National Epidemiologic Survey on Alcohol and Related Conditions-III: 10.4%) [96] and racial-ethnic differences reported in National Epidemiologic Survey on Alcohol and Related Conditions-III, one would expect 67.3% of individuals in a 12-month depression sample to be non-Hispanic White. The rate of White individuals represented in our study was statistically significantly different from 67.3% (χ^2^_1_=130.4, *P*<.001). When using lifetime prevalence rates, one would expect 72.5% of individuals with lifetime depression to be White. The rate of White individuals we found was significantly higher (χ^2^_1_=27.6, *P*<.001), though it was a relatively small difference (4.1%).

### Reporting of Other Group Demographics Outside of the United States

Out of the 46 articles conducted outside of the United States, only 25 (54%) reported on other demographic factors, such as language proficiency (n=13), country of residence (n=11), country of origin (n=5), migration status (n=2), and nationality (n=2). Most (n=11) of the studies that reported language only did so indirectly, as an inclusion criterion (eg, “participants must speak Dutch fluently”). Similarly, most (n=9) of the studies that reported the country of residence did so indirectly, (eg, “participants must reside in Sweden”). Some studies reported on multiple of the aforementioned demographics, therefore, there are more reported demographic variables (ie, 33) than the number of studies reporting these (ie, 25).

## Discussion

### Principal Findings

Our first aim was to examine the overall reporting of racial and ethnic diversity in RCTs of iCBT for depression. The second aim was to explore the representation of specific minoritized groups within the studies reporting race-ethnicity. Our search revealed substantial gaps in the literature. Out of the 62, only 17 (27%) of the papers included in this paper had data on race, and 12 (19%) included ethnicity. Focusing on the United States, even when race was reported, White individuals were overrepresented in samples relative to US census population estimates (61.6%) [97] and the expected estimate of non-Hispanic White individuals in depression samples (67.3%) [98].

### Limitations

Before interpreting our findings, several caveats are worth noting. First, we recognize that failure to report racial-ethnic identity information does not guarantee that racial-ethnic diversity is not well-represented in a trial's sample. However, we believe that it is unlikely that researchers are collecting very racially diverse samples and choosing not to report on that aspect of the sample composition. In some countries reporting race or ethnicity might be more limited by regulations like ethical review board approval. Nonetheless, only 25 (54%) out of 46 of studies conducted outside of the United States reported other characteristics such as nationality, country of origin, or migration status. Additionally, it is worth noting that it is not always clear what the implications of underreporting are. For example, in a large individual-patient data meta-analysis, Karyotaki et al [99] reported that individuals from racial-ethnic minoritized groups experienced poorer outcomes in guided iCBT than “native-born” participants. These findings are worth replicating as they imply that racial-ethnic groups may respond differently to iCBTs and are concerning regarding the underrepresentation of minoritized groups in iCBT research. Nonetheless, even if race-ethnicity was not a predictor of outcomes in treatment, the differential enrollment of participants from different racial-ethnic groups is itself a racial-ethnic disparity. We excluded gray literature such as posters and other unpublished works. While these exclusions represent a limitation of this study (ie, fewer RCTs included), it is not clear that including these would have changed our conclusions that the reporting of representation is poor in RCTs of self-help iCBT. Finally, we chose to focus on iCBT, given its popularity and potential to reduce the public health burden of depression [100,101]. However, self-help iCBTs are not the only low-intensity treatment available. For example, bibliotherapy (ie, printed self-help media) is also a low-intensity treatment that may be preferred by many individuals, with some evidence suggesting that some racial-ethnic minoritized groups may prefer bibliotherapy over digital interventions [23].

### Implications of Underrepresentation and Methodological Considerations

Our results suggest that the rate of reporting of ethnicity in iCBT studies in the United States has improved relative to the prior report by Polo and colleagues [11] for RCTs of face-to-face psychotherapy for depression. However, reporting of background characteristics in iCBT RCTs is relatively poor in Europe and elsewhere. Indeed, only 2 RCTs of iCBT for depression conducted outside of the United States reported race or ethnicity, one of which was a cultural adaptation. This finding is surprising given that an estimated 23 million non-European citizens (5% of the population) live in Europe [102]. This underreporting makes it challenging to determine how well our current interventions fit with the experiences of minoritized groups. While it is a possibility that logistic barriers such as internet access may be responsible for the lower representation of individuals from racial-ethnic minoritized groups in iCBT studies, it is unclear if these differences are large enough to account for the disparities in trial representation [103]. Ramos et al [29] noted that diversity, equity, and inclusion were not guiding principles in iCBT research. Similarly, our findings suggest a need to understand how individuals from racial-ethnic minoritized groups engage in iCBT research and how to increase that engagement.

Another explanation for the lack of reporting and representation could be group differences in the acceptability of iCBT (ie, internet-based self-help not being a preferred format). However, there is evidence that compared to non-Hispanic White adults, Asian, Hispanic, non-Hispanic Black, and other racial-ethnic groups report either equal or greater willingness to use and choose to learn about iCBTs. Thus, overall willingness to use iCBTs is unlikely to explain differences in initiating iCBT trials.

Furthermore, the relevance of race and ethnicity as terms in countries outside the United States warrants additional examination. It is essential to investigate alternative methods of describing samples that are appropriate for different countries while allowing for cross-country comparisons. The low number of iCBT studies focusing on minoritized individuals' mental health makes it challenging to advocate for the using of iCBT to reduce health disparities in these populations, especially outside the United States. Hence, it is essential to conduct research that encompasses diverse populations and reports on their characteristics.

### Future Directions

This lack of knowledge regarding race and ethnicity has implications for implementing iCBT programs globally. Providers, for example, worry about the lack of diversity represented in the literature since this could lead to engagement challenges [104]. Acknowledging that a primarily White sample does not represent the general population may be an essential step to improving engagement. Numerous efforts to culturally adapt interventions to increase cultural competence of clinicians have been made [105]. However, we still lack information on how these adaptations translate to a self-help format to reduce barriers to access for minoritized groups. Currently, hundreds of online mental health resources are available to the public, but it is sometimes unclear what they offer users [106]. Clinicians and clients have no easy way of knowing which resources are evidence-based and suitable for individuals from racial-ethnic minoritized groups. The label “CBT” (cognitive behavioral therapy) could be a way to identify evidence-based mental health services. However, it is hard to determine whether digital interventions apply CBT principles correctly and whether this delivery format is ideal for members of racial-ethnic minoritized groups.

The extent to which racial-ethnic minoritized groups, other than US non-Hispanic Black adults, use self-help iCBT has received little attention [11,29]. It has been more than a decade since the National Institutes of Health, CONSORT, and JARS guidelines [12,13,107] introduced criteria regarding race and ethnicity, and the outlook has not improved significantly. Although reporting of race has improved, it is not being reported to the extent that it could be helpful. Further, a potential future direction includes developing new guidelines similar to the CONSORT and JARS [13,107] for journals oriented toward internet-based mental health. These guidelines could involve (1) including racial-ethnic groups with a breakdown; (2) describing the type and intensity of support being provided for guided self-help; (3) ensuring that samples are representative of gender, racial-ethnic groups, and other underreported identities; and (4) detailing which elements of CBT are included in the protocol. This standardization could facilitate the implementation of similar protocols in hospitals, mental health foundations, and businesses interested in providing mental health services to a broader population. The lack of reporting could also be improved by increasing outreach efforts and ensuring racial-ethnic diversity is included in RCTs for self-help iCBT.

### Conclusions

There is a lack of reporting of racial and ethnic diversity in clinical trials of iCBT, and the representation of individuals from racial-ethnic minoritized groups is quite poor. This gap has significant implications for the generalizability of the findings currently in the literature, as these might not apply to individuals from racial-ethnic minoritized groups. Therefore, improving the representation of racial-ethnic diversity in trials of iCBT should be a key direction for the field going forward.

## Appendix

Multimedia Appendix 1 Search string from MetaPsy.

Multimedia Appendix 2 Search string for articles published from 2021 to 2022.

Multimedia Appendix 3 Risk of bias assessment for articles not included in MetaPsy (n=20).

## Abbreviations

CBT: cognitive behavioral therapy
CONSORT: Consolidated Standards of Reporting Trials
iCBT: internet-based cognitive behavioral therapy
JARS: Journal Article Reporting Standards
PRISMA: Preferred Reporting Items for Systematic Reviews
RCT: randomized controlled trial
ROB: Risk of Bias
