# Supplementary material for: Race, Ethnicity, and Other Cultural Background Factors in Trials of Internet-Based Cognitive Behavioral Therapy for Depression: Systematic Review
Source: J Med Internet Res. 2024 Feb 1;26:e50780. doi: 10.2196/50780 (PMC10870215; doi:10.2196/50780)
Supplement: Multimedia Appendix 2 [file jmir_v26i1e50780_app2.docx]

Appendix 2. Search string 2021-2022

**PubMed**

(Computerized OR internet OR web OR internet-based OR web-based OR app OR app-based OR online OR e-mail OR ehealth OR etherapy) AND Psychotherapy [MH] OR psychotherap*[All Fields] OR cbt[All Fields] OR "behavior therapies"[All Fields] OR "behavior therapy"[All Fields] OR "behavior therapeutic"[All Fields] OR "behavior therapeutical"[All Fields] OR "behavior therapeutics"[All Fields] OR "behavior therapeutist"[all Fields] OR "behavior therapeutists"[All Fields] OR "behavior treatment"[All Fields] OR "behavior treatments"[All Fields] OR "behaviors therapies"[All Fields] OR

"behaviors therapy"[All Fields] OR "behaviors therapeutics"[All Fields] OR "behaviors therapeutic"[All Fields] OR "behaviors therapeutical"[All Fields] OR "behaviors therapeutist"[All Fields] OR "behaviors therapeutists"[All Fields] OR "behaviors treatment"[All Fields] OR "behaviors treatments"[All Fields] OR "behavioral therapies"[All Fields] OR "behavioral therapy"[All Fields] OR "behavioral therapeutics"[All Fields] OR "behavioral therapeutic"[All Fields] OR "behavioral therapeutical"[All Fields] OR "behavioral therapeutist"[All Fields] OR "behavioral therapeutists"[All Fields] OR "behavioral treatment"[All Fields] OR "behavioral treatments"[All Fields] OR "behaviour therapies"[All Fields] OR "behaviour therapy"[All Fields] OR "behaviour therapeutic"[All Fields] OR "behaviour therapeutical"[All Fields] OR "behaviour therapeutics"[All Fields] OR "behaviour therapeutist"[all Fields] OR "behaviour therapeutists"[All Fields] OR "behaviour treatment"[All Fields] OR "behaviour treatments"[All Fields] OR "behaviours therapies"[All Fields] OR "behaviours therapy"[All Fields] OR "behaviours therapeutics"[All Fields] OR "behaviours therapeutic"[All Fields] OR "behaviours therapeutical"[All Fields] OR "behaviours therapeutist"[All Fields] OR "behaviours therapeutists"[All Fields] OR "behaviours treatment"[All Fields] OR "behaviours treatments"[All Fields] OR "behavioural therapies"[All Fields] OR "behavioural therapy"[All Fields] OR "behavioural therapeutics"[All Fields] OR "behavioural therapeutic"[All Fields] OR "behavioural therapeutical"[All Fields] OR "behavioural therapeutist"[All Fields] OR "behavioural therapeutists"[All Fields] OR "behavioural treatment"[All Fields] OR "behavioural treatments"[All Fields] OR "cognition therapies"[All Fields] OR "cognition therapie"[All Fields] OR "cognition therapy"[All Fields] OR "cognition therapeutical"[All Fields] OR "cognition therapeutic"[All Fields] OR "cognition therapeutics"[All Fields] OR "cognition therapeutist"[All Fields] OR "cognition therapeutists"[All Fields] OR "cognition treatment"[All Fields] OR "cognition treatments"[All Fields] OR psychodynamic[All Fields] OR Psychoanalysis[MH] OR psychoanalysis[All Fields] OR psychoanalytic*[All Fields] OR counselling[All Fields] OR counseling[All Fields] OR Counseling[MH] OR "problem-solving"[All Fields] OR mindfulness[All Fields] OR (acceptance[All Fields] AND commitment[All Fields] ) OR "assertiveness training"[All Fields] OR "behavior activation"[All Fields] OR "behaviors activation"[All Fields] OR "behavioral activation"[All Fields] OR "cognitive therapies"[All Fields] OR "cognitive therapy"[All Fields] OR "cognitive therapeutic"[All Fields] OR "cognitive therapeutics"[All Fields] OR "cognitive therapeutical"[All Fields] OR "cognitive therapeutist"[All Fields] OR "cognitive therapeutists"[All Fields] OR "cognitive treatment"[All Fields] OR "cognitive treatments"[All Fields] OR "cognitive restructuring"[All Fields] OR (("compassion-focused"[All Fields] OR "compassion-focussed"[All Fields]) AND (therapy[SH] OR therapies[All Fields] OR therapy[All Fields] OR therape*[All Fields] OR therapis*[All Fields]OR Therapeutics [OR treatment*[All Fields])) OR ((therapy[SH] OR therapies[All Fields] OR therapy [All Fields] OR therape*[All Fields] OR therapis*[All Fields] OR Therapeutics[MH] OR treatment*[All Fields]) AND constructivist*[All Fields]) OR "metacognitive therapies"[All Fields] OR "metacognitive therapy"[All Fields] OR "metacognitive therapeutic"[All Fields] OR "metacognitive therapeutics"[All Fields] OR "metacognitive therapeutical"[All Fields] OR "metacognitive therapeutist"[All Fields] OR "metacognitive therapeutists"[All Fields] OR "metacognitive treatment"[All Fields] OR "metacognitive treatments"[All Fields] OR "meta-cognitive therapies"[All Fields] OR "meta-cognitive therapy"[All Fields] OR "meta-cognitive therapeutic"[All Fields] OR "meta-cognitive therapeutics"[All Fields] OR "meta-cognitive therapeutical"[All Fields] OR "meta-cognitive therapeutist"[All Fields] OR "meta-cognitive therapeutists"[All Fields] OR "meta-cognitive treatment"[All Fields] OR "meta-cognitive treatments"[All Fields] OR "solution-focused therapies"[All Fields] OR "solution-focused therapy"[All Fields] OR "solution-focused therapeutic"[All Fields] OR "solution-focused therapeutics"[All Fields] OR "solution-focused therapeutical"[All Fields] OR "solution focused therapies"[All Fields] OR "solution focused therapy"[All Fields] OR "solution focused therapeutic"[All Fields] OR "solution focused therapeutics"[All Fields] OR "solution focused therapeutical"[All Fields]OR "solution-focussed therapies"[All Fields] OR "solution-focussed therapy"[All Fields] OR "solution-focussed therapeutic"[All Fields] OR "solution-focussed therapeutics"[All Fields] OR "solution-focussed therapeutical"[All Fields]OR "solution focussed therapies"[All Fields] OR "solution focussed therapy"[All Fields] OR "solution focussed therapeutic"[All Fields] OR "solution focussed therapeutics"[All Fields] OR "solution focussed therapeutical"[All Fields] OR "self-control therapies"[All Fields] OR "self-control therapy"[All Fields] OR "self-control therapeutics"[All Fields] OR "self-control therapeutical"[All Fields] OR "self-control therapeutic"[All Fields] OR "self-control training"[All Fields] OR "self-control trainings"[All Fields] OR "self control therapies"[All Fields] OR "self control therapy"[All Fields] OR "self control therapeutics"[All Fields] OR "self control therapeutical"[All Fields] OR "self control therapeutic"[All Fields] OR "self control training"[All Fields] OR "self control trainings"[All Fields]

AND

(Depressive Disorder[MH] OR Depression[MH]OR dysthymi*[All Fields] OR "affective disorder"[All Fields]OR "affective disorders"[All Fields] OR "mood disorder"[All Fields] OR "mood disorders"[All Fields] OR depression*[All Fields] OR depressive*[All Fields] OR "dysthymic disorder"[MeSH Terms])

Limits: RCTs
