# Supplementary material for: Race, Ethnicity, and Other Cultural Background Factors in Trials of Internet-Based Cognitive Behavioral Therapy for Depression: Systematic Review
Source: J Med Internet Res. 2024 Feb 1;26:e50780. doi: 10.2196/50780 (PMC10870215; doi:10.2196/50780)
Supplement: Multimedia Appendix 3 [file jmir_v26i1e50780_app3.docx]

Appendix 3. Risk of bias (ROB) assessment.

Figure 2. Risk of bias (ROB) assessment for papers that did not have a prior ROB assessment on METAPSY (N=20)

Note: Cuijpers et al had published a risk of bias on the other articles included in our search (n=42).

Figure 3. Risk of bias assessment for papers that did not have a prior ROB assessment on METAPSY with categorical information (N=20).

| **Unique ID** | **Study ID** | **D1** | **D2** | **D3** | **D4** | **D5** | **Overall** |  |  |  |
| --- | --- | --- | --- | --- | --- | --- | --- | --- | --- | --- |
| 14 | (Dahne, 2019) |  |  |  |  |  |  |  |  | Low risk |
| 15 | (Dahne, 2019) |  |  |  |  |  |  |  |  | Some concerns |
| 19 | (Flygare, 2019) |  |  |  |  |  |  |  |  | High risk |
| 20 | (Forand, 2017) |  |  |  |  |  |  |  |  |  |
| 21 | (Guo, 2020) |  |  |  |  |  |  |  | D1 | Randomization process |
| 24 | (Jelinek, 2020) |  |  |  |  |  |  |  | D2 | Deviations from the intended interventions |
| 25 | (Johansson, 2019) |  |  |  |  |  |  |  | D3 | Missing outcome data |
| 37 | (O'Moore, 2018) |  |  |  |  |  |  |  | D4 | Measurement of the outcome |
| 42 | (Reins, 2019) |  |  |  |  |  |  |  | D5 | Selection of the reported result |
| 44 | (Lobner, 2018) |  |  |  |  |  |  |  |  |  |
| 47 | (Salamanca-Sanabria, 2020) |  |  |  |  |  |  |  |  |  |
| 49 | (Schure, 2019) |  |  |  |  |  |  |  |  |  |
| 56 | (Kingston, 2020) |  |  |  |  |  |  |  |  |  |
| 49 | (Bucker, 2019) |  |  |  |  |  |  |  |  |  |
| 51 | (Stiles-Shields, 2019) |  |  |  |  |  |  |  |  |  |
| 57 | (Oehler, 2020) |  |  |  |  |  |  |  |  |  |
| 58 | (Pihlaja, 2020) |  |  |  |  |  |  |  |  |  |
| 60 | (Bur, 2022) |  |  |  |  |  |  |  |  |  |
| 61 | (Kramer, 2022) |  |  |  |  |  |  |  |  |  |
| 64 | (Zhao, 2022) |  |  |  |  |  |  |  |  |  |

Note: Cuijpers et al had published a risk of bias on the other articles included in our search (n=42).
